# Pleistocene Hominins as a Resource for Carnivores: A c. 500,000-Year-Old Human Femur Bearing Tooth-Marks in North Africa (Thomas Quarry I, Morocco)

**DOI:** 10.1371/journal.pone.0152284

**Published:** 2016-04-27

**Authors:** Camille Daujeard, Denis Geraads, Rosalia Gallotti, David Lefèvre, Abderrahim Mohib, Jean-Paul Raynal, Jean-Jacques Hublin

**Affiliations:** 1 « Histoire Naturelle de l’Homme Préhistorique » (HNHP, UMR 7194), Sorbonne Universités, MNHN, CNRS, UPMC, UPVD, Paris, France; 2 « Centre de Recherche sur la Paléobiodiversité et les Paléoenvironnements » (CR2P, UMR 7207), Sorbonne Universités, MNHN, CNRS, UPMC, Paris, France; 3 Max Planck Institute for Evolutionary Anthropology, Department of Human Evolution, Leipzig, Germany; 4 Dipartimento di Scienze dell'Antichità, Università di Roma La Sapienza, Via dei Volsci 122, 00185 Roma, Italy; 5 De la Préhistoire à l'Actuel, Culture, Environnement, Anthropologie » (PACEA, UMR 5199 CNRS), Université de Bordeaux, Pessac, France; 6 «Archéologie des Sociétés Méditerranéennes » (UMR 5140 CNRS), Université Paul Valéry-Montpellier 3, France; 7 Institut National des Sciences de l'Archéologie et du Patrimoine, Rabat, Morocco; University of Oxford, UNITED KINGDOM

## Abstract

In many Middle Pleistocene sites, the co-occurrence of hominins with carnivores, who both contributed to faunal accumulations, suggests competition for resources as well as for living spaces. Despite this, there is very little evidence of direct interaction between them to-date. Recently, a human femoral diaphysis has been recognized in South-West of Casablanca (Morocco), in the locality called Thomas Quarry I. This site is famous for its Middle Pleistocene fossil hominins considered representatives of *Homo rhodesiensis*. The bone was discovered in Unit 4 of the Grotte à Hominidés (GH), dated to c. 500 ky and was associated with Acheulean artefacts and a rich mammalian fauna. Anatomically, it fits well within the group of known early Middle Pleistocene *Homo*, but its chief point of interest is that the diaphyseal ends display numerous tooth marks showing that it had been consumed shortly after death by a large carnivore, probably a hyena. This bone represents the first evidence of consumption of human remains by carnivores in the cave. Whether predated or scavenged, this chewed femur indicates that humans were a resource for carnivores, underlining their close relationships during the Middle Pleistocene in Atlantic Morocco.

## Introduction

Large carnivores are present in almost all Plio-Pleistocene archaeological sites, and bone damage (tooth and cut marks) among the faunal deposits indicate their close coexistence with hominins [1–5]. The increase of meat and fat in the diet of early *Homo* (c. 1.9 My), not only had an impact on brain and molar size [6,7], but also on their eco-ethological relationships with other predators or scavengers. The infiltration of hominins into the carnivore guild may have resulted in different forms of interactions or co-evolutionary processes, including direct competition for resources as well as passive confrontations, dispersals, extinctions, partitioning of resources, dependency, commensalism and eventually domestication during the Late Pleistocene [8–13]. Changes in hominin subsistence strategies might be the result of these interactions. Meat providers or dangerous competitors, large predators are considered alternately as either facilitating, or reducing the success of the very first human dispersals [14–19]. Inversely, humans had significant role in the extinctions of various carnivores in North America at the end of the Pleistocene [20] or much earlier in the Lower Pleistocene of East Africa [12]. As another example, the drastic decrease of the sabre tooth cats and giant hyenas and the establishment of the modern-day carnivore guild in the Early Middle Pleistocene on both sides of the Mediterranean are contemporaneous with the first long-term hominin settlements [21–23,14,16]. At that time, hominins hunted in groups and relied on new effective weapons; these two improvements allowed them to slaughter larger gregarious preys and to handle encounters with dangerous competitors [24–29,9,10]. Still, this was a period of stiff competition between large carnivores and hominins and many Middle Pleistocene mixed assemblages containing materials modified by humans and a diverse array of carnivores are recorded (e.g. [30,31,11]).

Nevertheless, although during the Plio-Pleistocene hominins and carnivores shared the same landscapes and competed for resources and natural shelter, there is poor evidence of direct confrontation, such as serious or lethal bone damage, before the Upper Paleolithic, when carnivore hunting became widespread [32,33]. Two earlier cases have been reported: the SK 54 australopithecine cranium of Sterkfontein (South Africa) [1] and the CN42174b Neanderthal parietal fragment of Cova Negra (Spain) [34,35], which both bear two holes that match the spacing of the canines of a leopard and were described as representing large felids attacks. However, most of the purported cases of predation by carnivores on humans, or vice-versa, are mostly inferred from indirect data: taxa frequency, age of death, location of tooth-marks or cut-marks on the carcasses (primary or secondary access) [36–38]. Whether tooth-marks on human bones or cut-marks on carnivore bones, we can only identify consumption marks without confidently discriminating between hunted animals and scavenged carcasses. In Plio-Pleistocene sites, human remains showing tooth-marks are often associated with the rest of the fauna consumed by carnivores. Examples include, Swartkrans [1], FLK 22, FLK-NN1 and NN3 at Olduvai [39,40] in Africa. In the Middle and Upper Pleistocene of Eurasia, examples are more numerous including those of Zhoukoudian [41,42], Atapuerca (Sima de los Huesos) [43], Grotta Guattari [44,45], Rochelot [46,47], Rochers-de-Villeneuve [48], Grotte de la Tour [49], Les Pradelles [50], Gruta da Oliveira [51], and Grotte du Bison at Arcy-sur-Cure [52], among many others. Such associations may suggest either direct predation on humans or scavenging on their remains.

In the early Middle Pleistocene (up to c.500 ky) of North Africa, successful large predators such as the sabre-tooth cat *Homotherium*, lived alongside leopards and lion-sized felids. These large cats were associated with increasingly modern canids and hyenids (e.g., *Crocuta* and *Hyaena* replacing *Pliocrocuta*), which were effective hunters and carcass consumers [53]. At that time, hyenids largely dominated carnivore spectra in caves as in open-air sites, whereas large canids (*Lycaon* and *Canis*) remained rare, unlike in the European carnivore guild [14,15,19], jackals (*Lupulella*) and foxes being more common. All these carnivores alternately occupied the living spaces with hominins and exploited the hunted or scavenged resources brought by their competitors [54]. Once again, there is no convincing evidence of carnivore action on Middle Pleistocene human remains in North Africa, although this hypothesis was put forward for the Sidi Abderrahmane fossils in Morocco [55,56], whose discovery in a supposed hyena den led Biberson to hypothesize a contribution by this large carnivore. A newly recognized Middle Pleistocene human femoral diaphysis from the stratigraphic Unit 4 of the Grotte à Hominidés at Thomas Quarry I, in Casablanca, Morocco, is the first definite example of this kind.

## Site Description

### Stratigraphy and age

**Fig 1:**
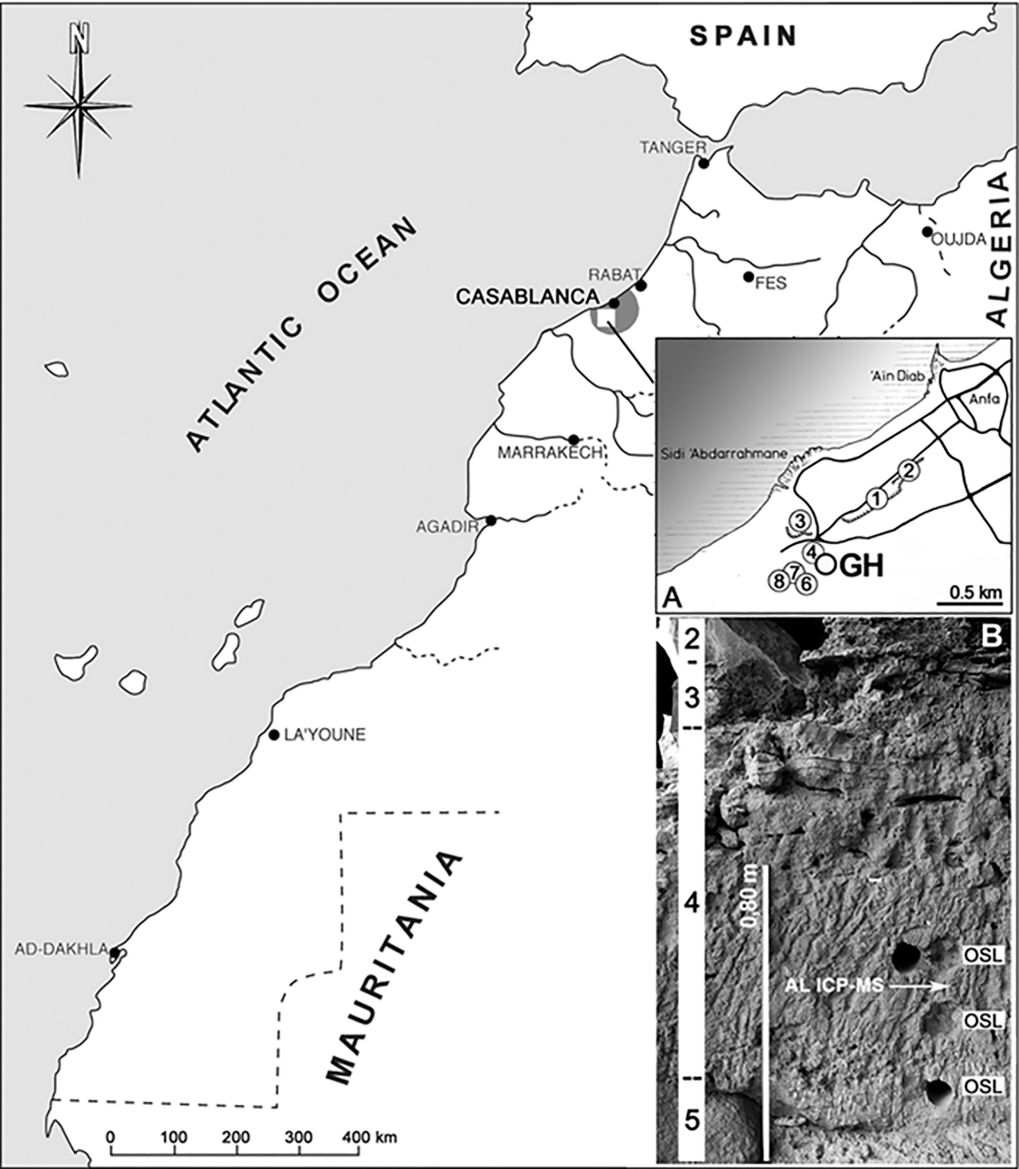
Location map (copyright JP. Raynal). A) Thomas Quarry I Hominid Cave (GH) and the main Lower Palaeolithic sites excavated at Casablanca: 1) Sidi Abderrahmane Grande Exploitation; 2) Sidi Abderrahmane-Cunette with Cap Chatelier and Grotte des Ours; 3) Sidi Abderrahmane-Extension; 4) STIC Quarry; 6) Thomas III Cave; 7) Thomas III “fissures”; 8) Oulad Hamida 1 Grotte des Rhinocéros. B) Stratigraphy in the eastern sector of GH and location of samples dated by OSL and laser ablation ICP-MS in Unit 4.

South-West of Casablanca, the locality of Thomas Quarry I was made famous after the discovery of a human half-mandible in a cave preserved in the northeastern wall of the quarry [57] (Fig 1A). First attributed to *Atlanthropus mauritanicus*, it was later assigned to *Homo rhodesiensis* [58] and associated with lithic artefacts and fauna [59]. From 1993 onwards, modern controlled excavations took place in the cave “Grotte à Hominidés” (GH), within the framework of the Franco-Moroccan co-operative project "Casablanca" [60,22].

GH is a large cavity that belongs to a complex paleo-shoreline cut into lower Midde Pleistocene formations [61–63]. Marine and overlaying continental sedimentary deposits preserved in GH (Fig 1B) are dated to the Middle and Upper Pleistocene by OSL [64], and based on lithostratigraphic [65,66,61,62] and biochronological evidences [67–69].

**Fig 2:**
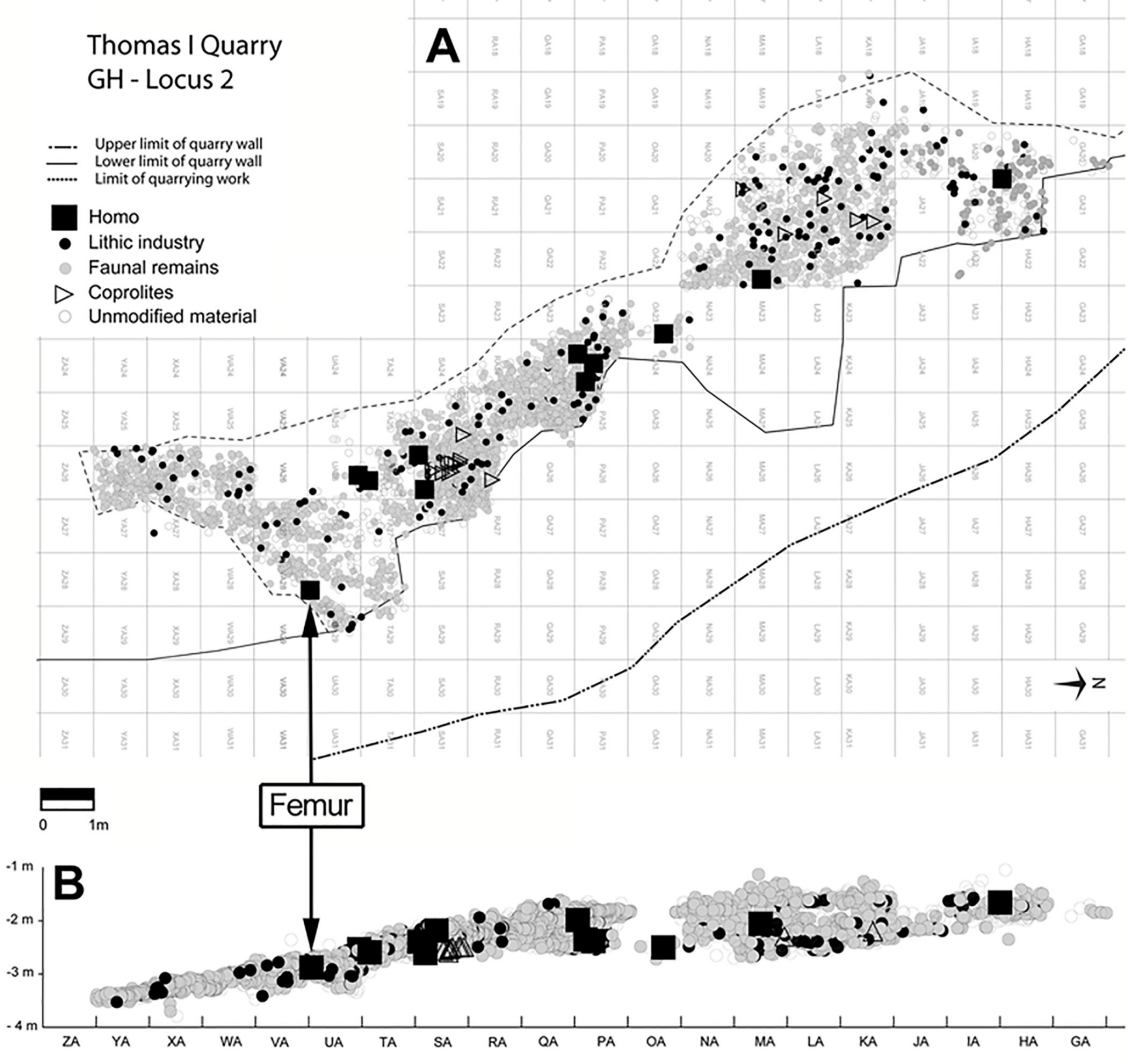
**Thomas Quarry I–GH distribution of finds in Unit 4: horizontal (A) and vertical (XZ) (B).** Vector restitution by R. Gallotti.

The Upper Pleistocene deposits show massive bedded rubefied sands with an abundant microfauna at the top (stratigraphic Unit 1), overlying a multilayer dripstone floor interbedded with loose red sands (stratigraphic Units 2 and 3). Stratigraphic Unit 4 is composed of bioclastic and quartzose well-sorted sands that mainly originated from reworked loose littoral deposits. Its detailed microstratigraphy reveals a complex history of sedimentary and post-depositional processes driven by semi-arid conditions. Unit 4 contains lithic artefacts, a rich mammalian macrofauna that is supplemented by the addition of a few reptiles and birds, and hominin fossils (Fig 2) [70,22,60,65,66]. Without any apparent discontinuity, Unit 4 rests on plurimetric collapsed blocks of older calcarenites imbedded in coarse sands and calcirudite with a coarse coquinoid matrix that form stratigraphic Unit 5. These facies, connected to a paleoshoreline with cliff and deep cavities cut into older formations, record a sea level highstand that predates OIS 15 [61,62].

Between 1994 and 2011, several new hominin fossils were recovered from stratigraphic Unit 4. In addition to the femur described below, they consist of several isolated teeth, cranial fragments, vertebrae, a complete mandible of an adult and a partial mandible of a young child. Laser ablation ICP-MS dating combining ESR and U-series data for modelling U-uptake has given a US/ESR age of 501^+94^_76_ ka for a human premolar, while OSL measurements indicate an age of circa 400 ky for embedding sediments. Nevertheless, biochronological and lithostratigraphic evidence point to a greater antiquity, closer to the base of the Middle Pleistocene [65,66].

### Human activity

A lithic assemblage has been recovered by recent excavations in the GH stratigraphic Unit 4, whose thickness ranges from 20 to 100 cm over an area of fifty square meters in the central and eastern parts of the cave (Fig 2). It is mainly manufactured from various quartzites as well as on a few flint nodules. The assemblage consists of 940 cobbles and pebbles (whole and broken) and 294 lithic artefacts. It is similar to the series collected at the time of the discovery of the first hominin fossil in 1969 [67]. Apart from a bifacial *chaîne opératoire*, which was processed outside the excavated area to obtain large cutting tools and heavy pointed tools, the other components indicate that lithic production mainly took place in the cave [65,66].

Unit 4 results from sedimentary processes that may have mixed artefacts with bones previously accumulated by predators. The spatial distribution of the finds do not appear to differ from a uniform model (Fig 2). This can be expected in the case of archaeological and/or paleontological concentrations disturbed by natural agents, but not in the case of *in situ* assemblages. However, within the archaeostratigraphic Unit 4, the absence of horizontal or vertical sorting of artefacts by weight and the presence of refittings of lithic objects seem to attest to at least a partial lithic production *in situ*.

### Fauna

In the faunal spectrum, gazelles and other antelopes are dominant among herbivores and a middle-sized jackal, *Lupulella mohibi*, dominates the carnivore assemblage [71,54]. Other carnivores include: hyenas (*Crocuta crocuta* and *Hyaena hyaena*), bears (*Ursus bibersoni*), leopards (*Panthera* sp.), wild cats (*Felis* cf. *libyca*), seals (*Monachus* sp.) and honey-badgers (*Mellivora capensis*). Porcupines (*Hystrix* sp.) are also present, as well as large gelada baboons (*Theropithecus oswaldi*). The dominant forms of bovids, gazelles (*Gazella* cf. *atlantica*) and alcelaphins (including the extinct genus *Parmularius* and the wildebeest *Connochaetes*) which represent 89% of the NISP for all bovids, both indicate an open environment, as does the occurrence of Hippotragini (*Oryx* sp.) and, among Perissodactyls, of the zebra *Equus* cf. *mauritanicus* and of the rhino *Ceratotherium* sp. Other ungulates are: suids (the extinct *Kolpochoerus* sp. and the modern form *Phacochoerus africanus*) and other rare bovids, including Bovini (*Bos* or *Pelorovis*) and Reduncini (*Redunca* sp.). The occurrence of the ground-dwelling giant gelada baboon and the absence of any other monkey provide an even greater indication of generally poor tree cover [72,53,54,65,66].

In an early study, carnivores were considered as the primary agent for bone accumulations and modifications [73], and this has been confirmed by a new taphonomic study of the large fauna recently excavated from Unit 4 [54,65,66]. Carnivores represent about 30% of the MNI. Coprolites are numerous. Large bovids, equids, rhinocerotids and suids have an attritional mortality and represent the majority of isolated teeth and autopodial elements, suggesting gathering activities over scavenged carcasses. On the other hand, small and middle-sized prey (bovid sizes 1 to 3) [1], like gazelles and alcelaphins, are represented by all age classes and by a majority of limb bones, suggesting predation with selective transportation of elements. The proportion of tooth-marked bones (20% of recorded and legible remains) and the homogeneous distribution of marks on the long bones suggests that the carcasses were primarily consumed by carnivores. The dimensions of tooth-marks and coprolites indicate that different sizes of carnivores were gnawing bones and denning in the cave. Porcupines also left some gnawing marks. Despite the association of the faunal remains with lithic artefacts and human remains, no cut-marks were found, which questions the role of humans in the bone accumulations. However, as the studied assemblage comes from the deepest part of the cave, human occupation could have been concentrated closer to the entrance in a zone that has not been excavated yet or has been destroyed by quarrying.

## Material and Methods

The studied ThI94-UA28-7 femur is housed in the Institut National des Sciences de l'Archéologie et du Patrimoine–INSAP (Madinat Al Irfane, Rabat, Morocco). Access is permitted under Moroccan regulations. The present study, which complies with all relevant regulations, was authorized by permit n°146 of October 2012 issued by the Ministère de la Culture of the Kingdom of Morocco.

### Femur description

Following [74], the biomechanical length is the average of the distances parallel to the diaphyseal axis of the femur from the proximal extension of the diaphyseal axis on the superior neck, just medial to the greater trochanter, to each distal condyle. A photo taken perpendicular to the cross section was digitized four times, enlarged about 10 times on a Summagraphics 1812 tablet, and the values averaged. The cross section of the diaphysis was analysed using the parameters defined by [75] who summarized many earlier papers and provided synthetic comparisons.

### Taphonomy

We observed the bone surface with the naked eye and with a stereomicroscope (x20) under low magnification. We recorded types and locations of relevant modifications on the outer surface, including those made by rodents, carnivores or hominins as well as chemical and mechanic modifications [76–79]. We classified each of the carnivore marks as being one of the following types of damage [80–82,77]: pits (shallow depressions whose bottom is compact bone), punctures (deep holes whose bottom is cancellous bone), scores (longitudinal and parallel shallow scratches whose bottom is compact bone and usually running perpendicular to the longitudinal axis), furrows (deep and wide grooves with irregular margins whose bottom is cancellous bone tissue), notches (semi-circular removal due to the puncturing of the bone) or corrosion by gastric acids (corroded and polished surfaces and edges). Pits and punctures are circular or elongated (at least twice as long as wide). Scores are considered here as tooth marks that are about three times longer than wide. Regarding tooth-mark measurements, we focused on pits (maximal length and breadth), taking into account the tissue location (cancellous bone from epiphyseal sections and dense cortical bone from diaphyseal sections) [83,84] and we used actualistic data for comparative purposes [85–87,83]. The identification of the type of breakage (ancient green or dry bone breakage or recent breakage) was made based on the fracture colour, shape, feature and angle, as well as on associated marks [88,89].

## Results

### Description of the human femoral diaphysis (THI94-UA28-7)

**Fig 3:**
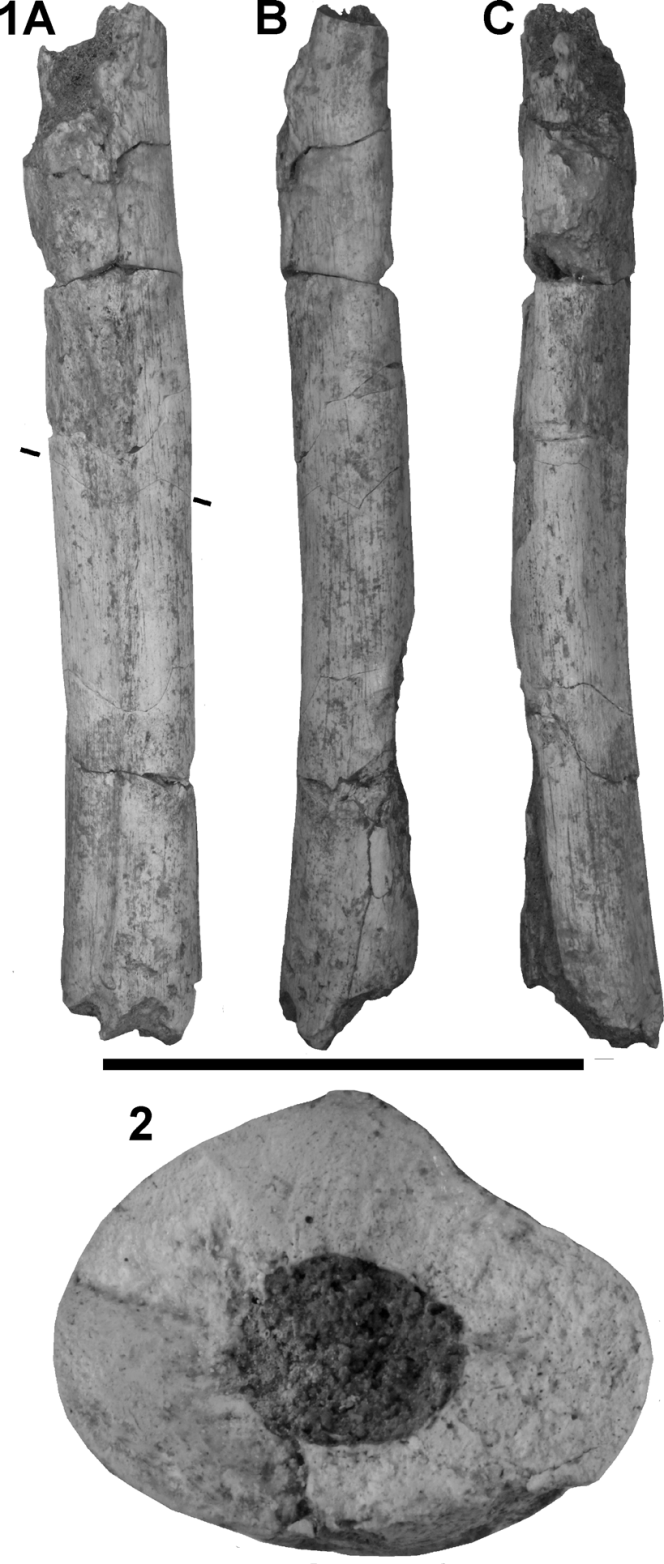
**The femoral diaphysis ThI94-UA28-7 views:** 1A) posterior, 1B) medial and 1C) lateral; 2) cross-section at the level indicated in 1A. Scale bar = 10 cm for 1) and 2.5 cm for 2). Photos by D. Geraads.

ThI94-UA28-7 (Fig 3) is the partial shaft of a left femur of adult size (length of the preserved part 218 mm). At the proximal end, it is irregularly broken at the distal portion of the lesser trochanter, and at the distal end, about 2 cm distal to the beginning of the divergence of the two components of the *linea aspera*. The bone is too incomplete to even roughly estimate its total length. The bone surface is well-preserved on most of the posterior and medial, and part of the lateral faces, but some cortical bone is missing on most of the anterior face. Neither the external aspect, nor that of the internal structure, which was examined at two different levels before the shaft segments were glued back together, show evidence of crushing. In medial view, the imperfectly preserved anterior border of the bone is virtually straight and the better-preserved posterior border confirms that the curvature of the shaft was certainly weak. In posterior view, the medial outline is slightly convex, and the lateral outline slightly concave. Variations in transverse diameter are slight, but the smallest section is probably located close to the mid-length of the complete bone; there is no evidence that it was distally located, as in many early *Homo* [90,91]. Not enough of the bone is preserved to estimate the degree of anteversion, but it was certainly low.

Except at the distal end, the cross section is distinctly compressed antero-posteriorly, especially proximally, but there is no medial-localized swelling below the missing neck. Unfortunately the lateral part of the bone is not preserved at this level, preventing estimates of the sub-trochanteric platymeric index. At about 55–60% of the biomechanical length (see Methods), at a level where the outline of the cross-section is virtually complete and the whole shaft is well preserved, the transverse diameter is much greater than the antero-posterior one, giving a very low pilastric index close to the minimum recorded values for modern humans, Middle Pleistocene hominins, as well as for early *Homo* (Table 1).

**Table 1:** Measurements of the ThI94-UA28-7 Femur and comparative data. Measurements at mid-length (ThI94-UA28-7 at about 60% of biomechanical length). 1: Trinil, ZhouKouDian, Berg Aukas, Gesher Benot Yacov, Kresna11, Tabun Ea, OH28; 2: KNM-ER-737, KNM-ER-803, KNM-ER-1481, BOU-VP-2/15, BOU-VP-19/63 (data from [92–96,90]). Measurements of Bouri femora calculated from figures in Gilbert [91].

	ThI94-UA28-7	Aïn Maarouf	Middle Pleistocene^1^ range (mean)	Early *Homo* East Africa^2^ range	Recent (N = c.1000) range
Shaft, A-P (M6)	23.2	26.2	22.1–35.9 (26.4)	21.7–29.3	18–40
Shaft, M-L (M7)	28.2	26.7	24.6–31.5 (28.5)	22.2–33.4	18–35
Pilastric index	82.2	98.1	79–114 (93)	82–88	79–146

The lines and ridges that mark the attachments of the hip muscles on the posterior face of the proximal end can hardly be traced. There is neither a distinct gluteal ridge, nor any evidence of a hypotrochanteric fossa, but what is preserved of the gluteal zone is a rough area whose medial margin, which provided attachment for the *m*. *adductor magnus*, is quite clear and centrally located on the shaft. Distally, it proceeds in a straight line to the *linea aspera*; proximally, this margin is so clearly marked that it would probably have extended to the missing lesser trochanter, as there is no evidence of an inflexion towards the medial side. In modern humans this inflexion leads to the spiral line for attachment of the *m*. *vastus medialis*; here, although the surface of this area is well preserved, the spiral line is absent.

Distally, the *linea aspera* remains centrally located; it is simple, slightly protruding and, although it remains bordered laterally by a shallow depression, there is no indication of a pilaster. The pilaster is always present in modern humans, but rare in early *Homo*, although occasionally present (e.g. in BOU-VP-19/63: [91], and probably Dmanisi: [97]). The *linea aspera* splits distally into the supra-condylar ridges, but nothing can be said about the popliteal surface.

The internal structure of the cross-section of the shaft could be examined on natural breaks before the segments were glued together (Fig 3: 2). The most informative section is located at a level where only a thin layer of bone is missing on the anterior face, so that the complete section can be reconstructed with reasonable accuracy. It is of course impossible to precisely determine the position of this section relative to the complete bone, but it must be close to 60–65% of the biomechanical length and comparisons should therefore be made mostly with data at 65%, but also at 50%.

The total subperiosteal (TA) and cortical (CA) areas of the cross section of ThI94-UA28-7 are TA = 483 mm², and CA = 401 mm² respectively. The value of the anteroposterior and mediolateral second moments of area, which characterize the resistance to anteroposterior and mediolateral bending stresses, are Ix = 13380 mm^4^ and Iy = 24869 mm^4^ respectively. Due to the reconstruction of a thin layer of bone on the anterior surface, and to the fact that the photo is only a projection on a plane of surfaces that are at different levels, these values carry some imprecision, but they do provide a basis for comparison.

The cortex provides resistance to axial load, and it is commonly assumed that Middle Pleistocene *Homo* had a relatively greater cortical area than modern humans, but this is mostly true of the distal portion [98,75]. However Trinkaus and Ruff [75] noticed that it also depends on the deposition/resorption process during development, and that the ratio CA/TA did not change much during the Pleistocene. ThI94-UA28-7 is well above the mean of Upper Pleistocene *Homo* at 50% of the biomechanical length, but close to the mean values for all groups at 65%, and within the ranges of all groups at both levels.

**Fig 4:**
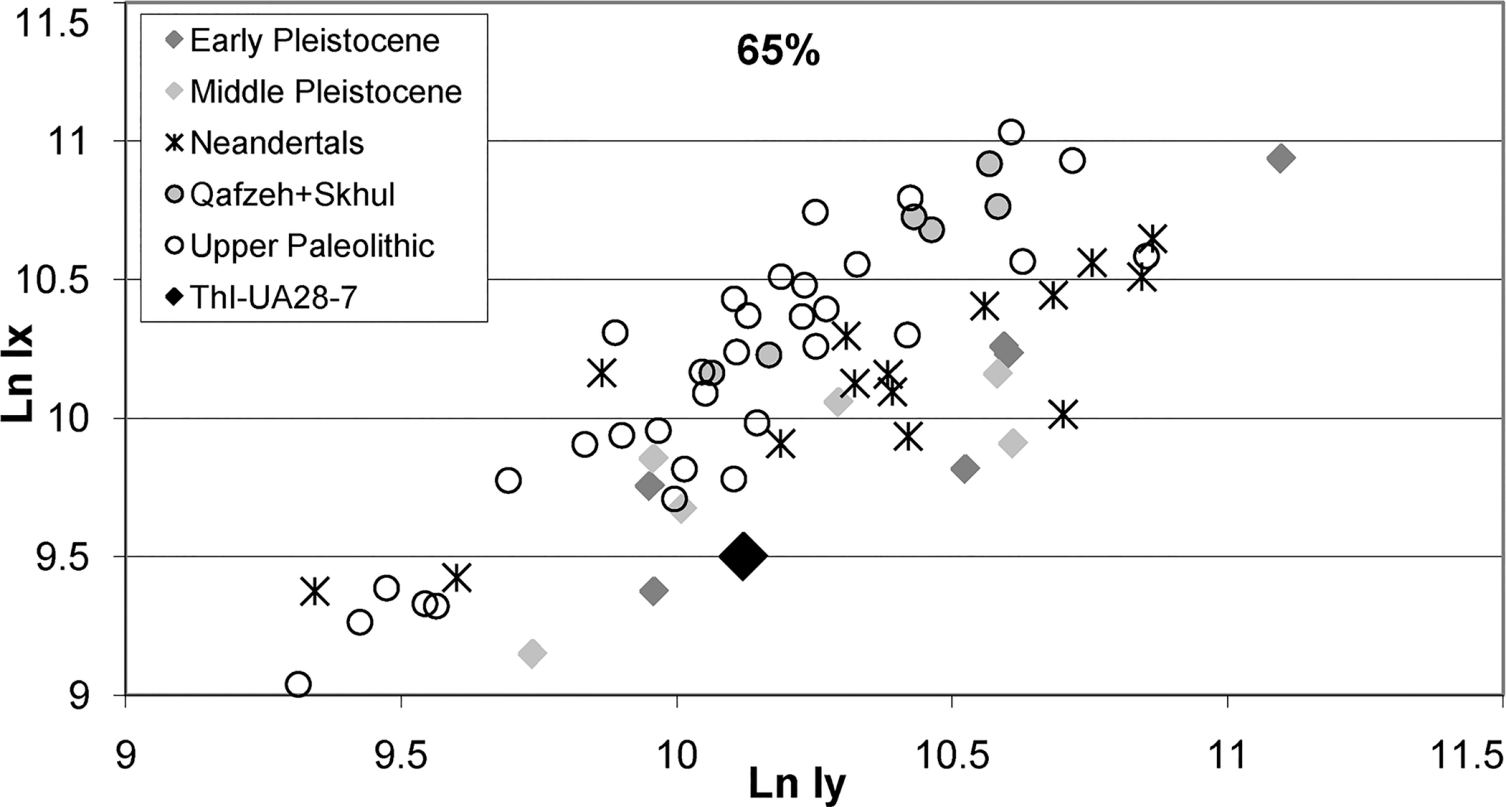
Comparative data taken from Trinkaus and Ruff [60] at the 65% level. Plot of transverse (Iy) vs. antero-posterior (Ix) second moments of area of ThI94-UA28-7 calculated by E. Trinkaus at level of Fig 3 (2). Scales in natural logarithms. Graph elaboration by D. Geraads.

Trinkaus and Ruff ([75] and references therein) observed that when the anteroposterior second moment of area (Ix) is plotted against the mediolateral one (Iy), there is a clear distinction between Lower/Middle Pleistocene *Homo* and later modern humans, with the potential resistance to medio-lateral stresses being greater in the former group. When added to their graphs, ThI94-UA28-7 plots very clearly with other Middle Pleistocene *Homo*, and far from later modern humans, both at the 50% and 65% distances (the latter is shown on Fig 4; ThI94-UA28-7 is still more distinct from modern humans at 50%). According to Trinkaus and Ruff [75], this difference has to do with pelvic and crural body proportions rather than with locomotor patterns.

On the whole, the GH diaphysis fits well within the group of known early Middle Pleistocene hominins. Among those that are geographically and chronologically close, it resembles the specimen from Aïn Maarouf [95] in the lack of pilaster, but otherwise differs by its lesser curvature, greater platymery, and relatively thicker cortical bone, but individual variability could account for all of these differences. The large femoral series from Atapuerca (Sima de los Huesos) [99,100] also display similar features with a lack of pilaster, medio-laterally expanded section, and thick cortical bone. Published anterior views ([99]: Fig 2) show that the minimum transverse diameter is located near mid-length, instead of more distally as in early African *Homo*, but more specimens need to be discovered at Casablanca before any meaningful comparisons can be made.

### Taphonomic analysis

ThI94-UA28-7 shows no evidence of significant mechanical or chemical alteration. Surface cracking and flaking is minimal (stage 1 of weathering), allowing good legibility. Some small corrosion cups can be noticed, but no trampling striations or cut-marks are present. Some smooth, linear, parallel grooves, likely vascular grooves, run along the surface.

**Fig 5:**
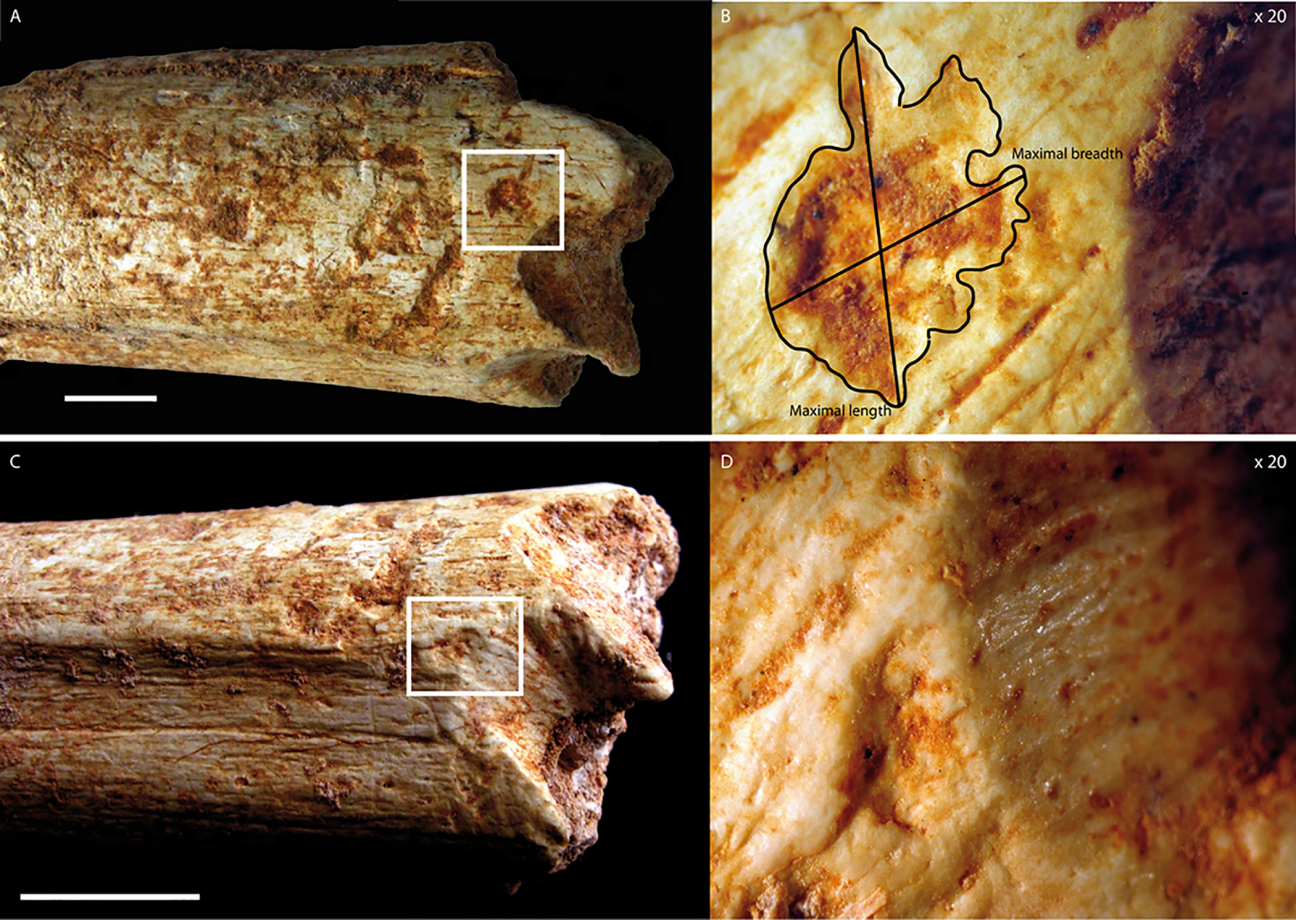
**Carnivore marks on the distal end of the Femur:** A) tooth-pits (scale = 1 cm); B) with maximal length and breadth indicated (X 20); C) notch (scale = 1 cm); D) with the magnification of the associated pit and micro-grooves (X 20). Photos by C. Daujeard.

**Fig 6:**
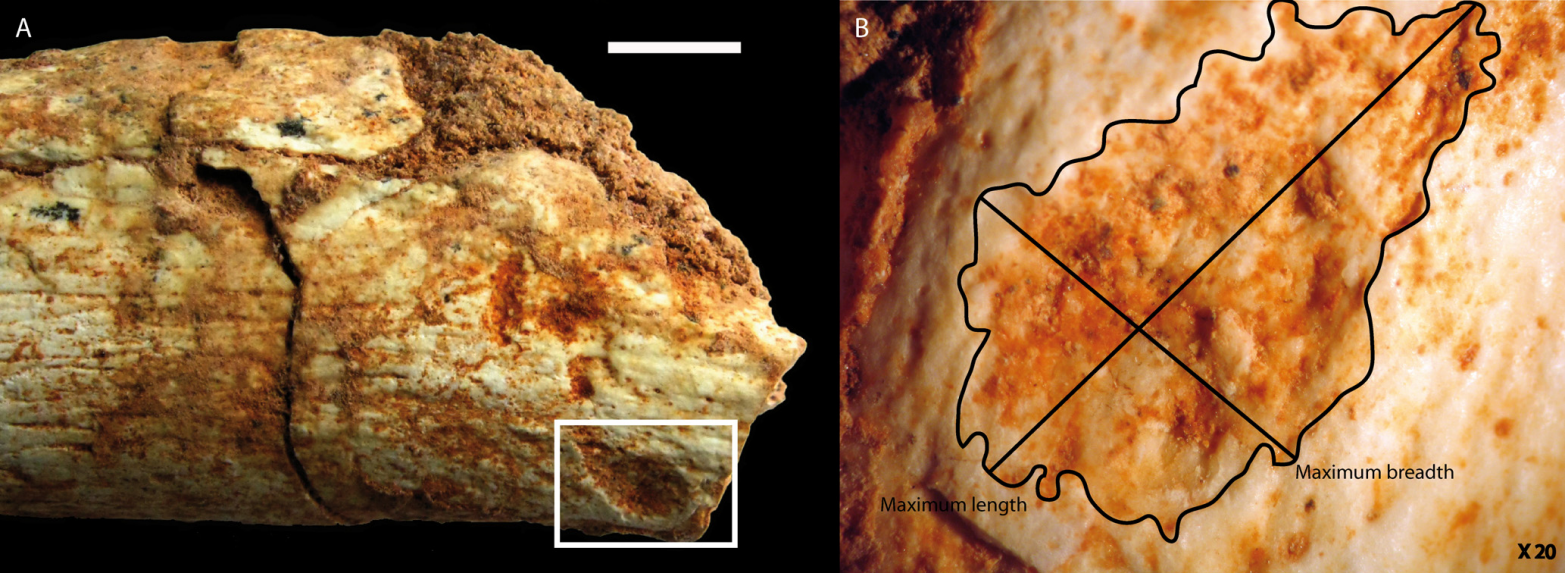
**Carnivore marks on the proximal end of the Femur:** A) tooth-pits (scale = 1 cm); B) with maximal length and breadth indicated (X 20). Photos by C. Daujeard.

Many carnivore tooth-marks were identified at both ends of the diaphysis. These marks were covered by sediments, indicating their antiquity. Magnification highlights the crenulated and polished appearance of the depression edges, characteristic of carnivore tooth-marks (Figs 5 and 6). They are all clustered at the two extremities of the bone, near the fracture edges. None is present in the central part. However, they are located on a portion where the compact bone is still thick, therefore considered to be tooth-marks made on dense cortical bone. They consist of circular or elongated pits, scores and notches. Nine circular pits, five scores and two notches are present on the distal portion, which is the most-chewed portion (Fig 5), and four pits (three circular and one elongated) were counted in the proximal portion (Fig 6). The largest pit dimensions are observed on the proximal section of the bone (Table 2). The means of the maximal length and breadth are 3.66 and 2.52 mm respectively.

**Table 2:** Measurements of carnivore tooth-marks. Measurements and means of the maximal length (L) and breadth (B) of the carnivore tooth-marks (P = pits; C = circular; L = elongated; S = Score; N = notch; SD = Standard Deviation; 95% C.I. = 95% confidence (two-tailed) interval; r = Pearson’s coefficient and p = associated probability).

Pit measurements (mm)	Length	Breadth
**Proximal shaft end**		
P1 C	6.8	3.8
P2 L	5.2	2.3
P3 C	2.2	1.3
P4 C	2.5	2.2
**Distal shaft end**		
P1 C	3	2.7
P2 C	3.8	2.3
P3 C	3.6	2.9
P4 C	2.3	2.1
P5 C	3.4	2.8
P6 C	2.8	2.5
P7 C	4	2.7
P8 C	4	2.9
P9 C	4	2.3
S1	6	1.7
S2	9	2.5
S3	10	2.9
S4	6.7	1.8
S5	5.6	1.7
N1	-	8
**Means pits (n = 13)**	**3.66**	**2.52**
Min-Max	2.2–6.8	1.3–3.8
95% C.I.	3,31–4,01	2,36–2,68
SD	1.26	0.58
r	0,72 (p = 0,006)	

**Fig 7:**
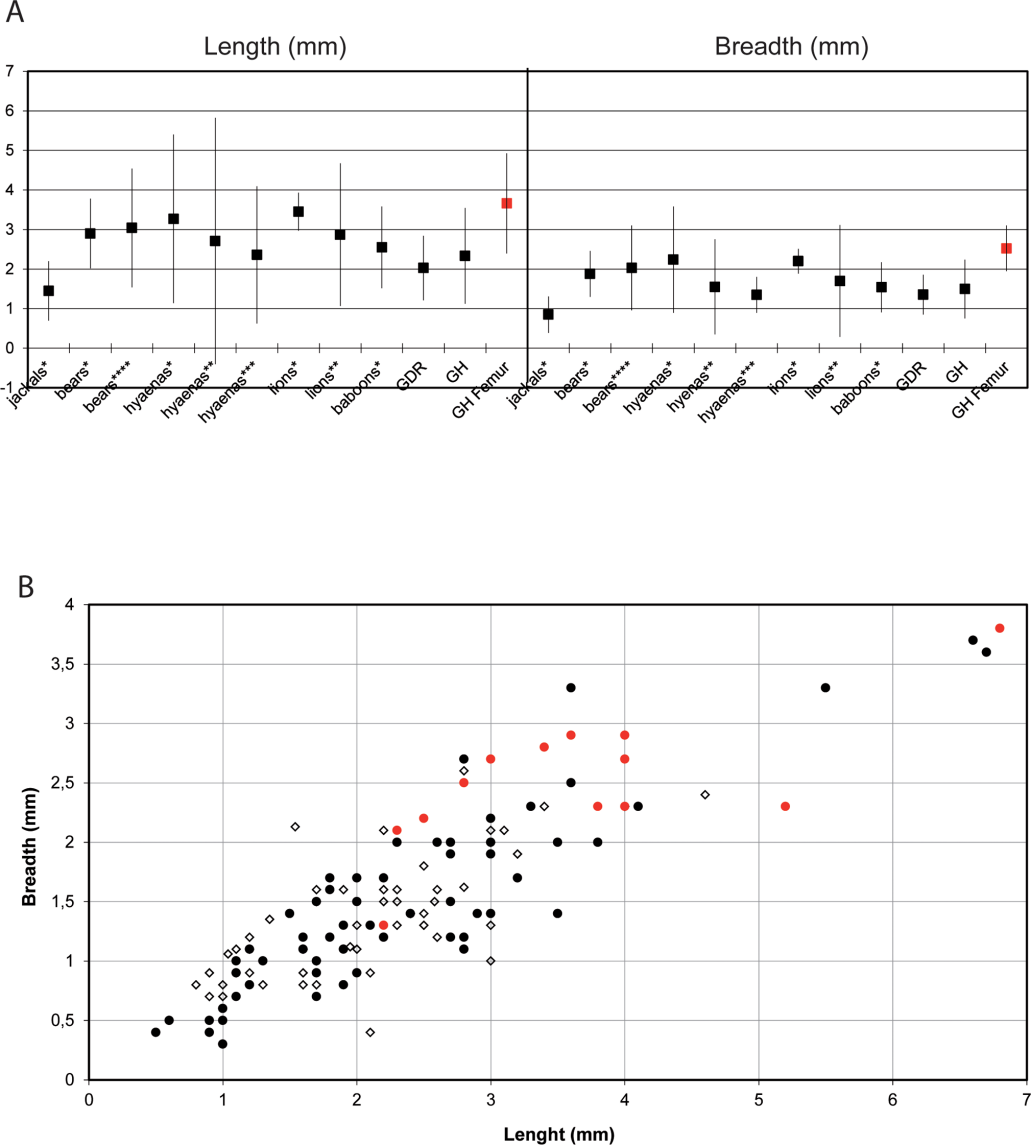
**Measurements of carnivore tooth-marks:** A) mean percentages and S.D. of pit sizes on cortical bone (mm). For comparative purposes, samples with (*) have been taken from Domínguez-Rodrigo and Piqueras [83]; samples with (**) from Andres et al. [86]; samples with (***) from Selvaggio and Wilder [85] and samples with **** from Saladié et al. [87]; B) maximal length and breadth (mm) for pit marks on cortical bone at GDR (n = 59: white diamond), GH (n = 70: black circles) and on the GH human femur (n = 13: red diamond). Graph elaboration by C. Daujeard.

Actualistic data document the strong overlap of pit dimensions created by carnivores of different sizes, specifically tooth-marks on cortical bone [101–103,80,83,85–87]. Even so, given most of these data, the presence on ThI94-UA28-7 of five pits larger than 4 mm in length and 2 mm in breadth and the size range and means of all pit dimensions indicate that they are attributable to a large carnivore (Fig 7A). Given the faunal list of carnivores present in GH–Unit 4 or elsewhere at that time in North Africa, hyenas (*Crocuta crocuta* or *Hyaena hyaena*), bears (*Ursus bibersoni*) or a large-felid such as the lion-sized *Panthera* sp. or the sabre-toothed cat *Homotherium* (unknown at GH but present at Tighenif c. 800 ky), may have partly eaten this bone. Pit measurements for bears usually fall between those of medium and large-sized carnivores (Fig 7A), making it a potential bone marker in this case. Moreover, Arilla et al. [104] showed that some small young starving bears could generate tooth-marks as large as those of old individuals or other large carnivores in modern samples.

**Fig 8:**
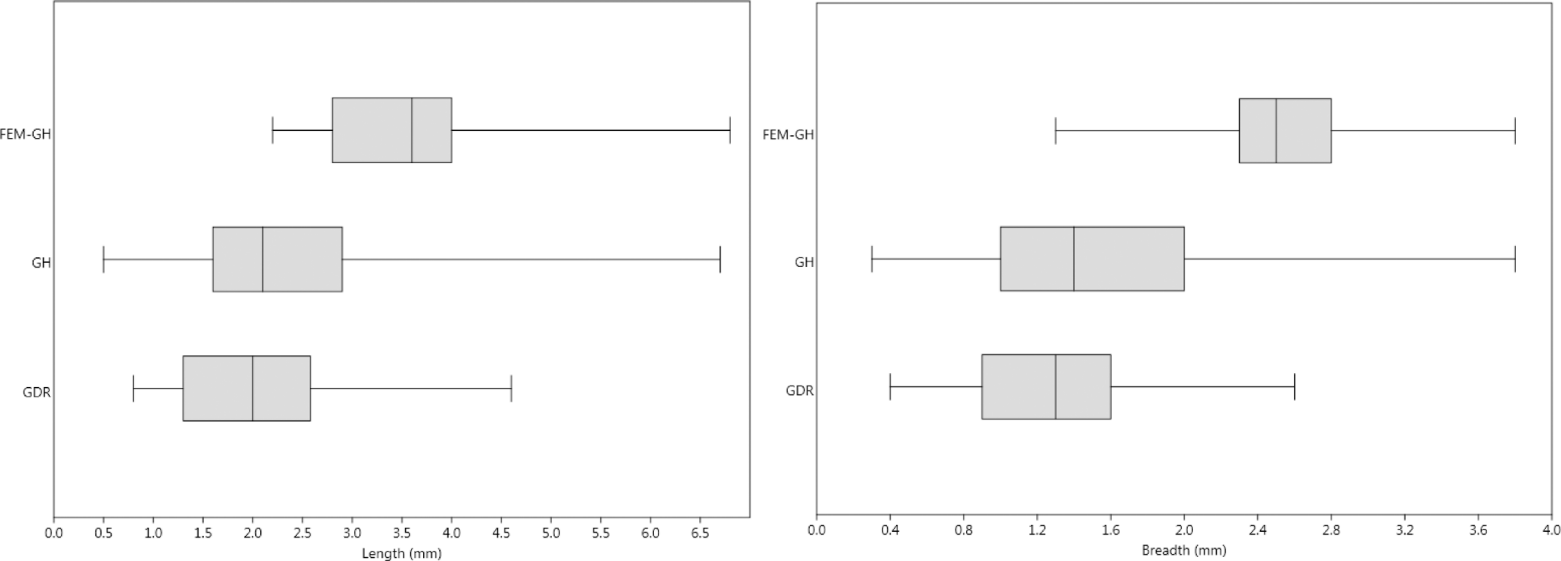
Distribution of the pit dimensions (length and breadth) observed on cortical bones at GH, GDR and the GH human femur (median, 25–75 percent quartiles and minimum and maximum values). Graph elaboration by C. Daujeard.

We also compared pit measurements of the femur with those left on other cortical bone fragments of GH and Grotte des Rhinocéros (GDR) (Figs 7B and 8). GDR is an Acheulean cave located very close by (0.5 km), with sub-contemporaneous levels that provide a large sample of tooth-marked bones. Both series have the same carnivore taxa (*Lupulella*; *Panthera*; *Hyaena*; *Crocuta*; *Ursus*; *Mellivora*; *Felis*) [53,54,68]. Similarly, the middle-sized canid is dominant. The pit dimensions do not show significant differences between the whole samples of GH and GDR (Kruskal-Wallis test, p>0.05; from [105,106]), whereas there are significant differences between the pit dimensions of the femur and those of GH and GDR (Kruskal-Wallis test, p<0.05; from [105,106]). The size ranges of the pits are large, confirming that bone-eaters of various sizes were using the two caves. The dominant middle-sized canid *Lupulella*, associated with small felids and mustelids, must be responsible for the main group of pits whose dimensions do not exceed 3.5 mm. In contrast, the dimensions (lengths and breadths) of the femur pits clearly belong to the largest ones present in the two caves, even exceeding the upper limit of the size ranges and reaching those attributable to hyenids, large felids or ursids (above 4 mm long and 2 mm broad).

Two notches and green bone fractures with polished edges were recorded on the distal extremity. One of the two notches has a breadth of 8 mm (Table 2) and shows micro-grooves on its fracture plane. Its polished edges and association with a carnivore pit indicate that this bone was chewed while it was still fresh (Fig 5). On the proximal extremity, one of the fractures may be associated with pits and may also be due to a perimortem breakage. In contrast, the longitudinal orientation and the rough appearance of the other fracture indicate a post-mortem breakage (on dry bone).

By crushing the distal and proximal epiphyses for the consumption of the red marrow, the carnivore reduced the long bone to an almost complete shaft cylinder which is ragged at the ends ([1]: p.71; [62]: p.51; [64]: p.325). The gnawing stopped at that stage of the cylinder, i.e. stage 5 of bovid femur consumption by wolves [82]. The rounded polished appearance of the edges at both ends of the medullar cavity may have resulted from the carnivore repeatedly licking into it, rather than attempting to crush the diaphysis, a more common and more efficient way of extracting marrow [77]. Green bone fractures, chewing, and marrow-recovering confirm consumption soon after death.

## Discussion

### Who were the culprits?

According to modern data [107–111,80–83,104], large felids and ursids are less efficient long-bone crackers than hyenids or canids. Marks left by felids are typically focused on the softer parts of the appendicular elements, mainly on the humerus and femur articular portions, but without fully destroying them and leaving few tooth-marks (mostly scores, furrows and crenulated edges). In contrast, hyenas are able to remove the two articular portions and break the diaphysis of a large mammal femur which has been carried to their den, generating abundant bone cylinders and leaving numerous tooth scratches and pits on the fragments surfaces [112–114,81]. In the case of the Rochers-de-Villeneuve [48] and Grotte de la Tour human femora [49], possibly gnawed by a cave hyena, articular portions were also removed. Similarly, tooth-marks are clustered at the two ends with more intense activity near the distal fracture, but diaphyseal fragments were more reduced than that of GH, with the production of a “channeled bone” by removal of substantial longitudinal flakes.

The circumference of the GH femur diaphysis is still virtually complete and there is no evidence of bone splintering in the manner of hyenas, indicating that marrow extraction activity might have been interrupted. However, the softer parts have been completely crushed and the dense cortical portion has been reached on both sides, which is not consistent with the modest action of felids or even ursids. The latter usually leave part of the two articular portions moderately furrowed and cause some perpendicular and sharply incised tooth scores in the compact tissue of the diaphysis extremities [43,81,109]. Both types of marks are absent on ThI94-UA28-7.

While tooth-mark measurements do not allow the identification of one potential consumer of the femur, the shaft reduction stage clearly indicates a hyena as the most likely candidate (*Crocuta crocuta* or *Hyaena hyaena*). In this regard, we may notice that many Plio-Pleistocene human remains have been discovered in caves used as hyena dens (cf. *Crocuta crocuta*, *Pachycrocuta bellax*, *Parahyaena brunnea*, *Chasmaporthetes nitidula* and *Chasmaporthetes silberbergi* at Sterkfontein Valley in South Africa [1,84]; *Pachycrocuta brevirostris* at Zhoukoudian in China; *Crocuta crocuta* at Wezmeh Cave in Iran [115]; *Crocuta crocuta spelaea* at Grotta Guattari in Italy; Gruta da Oliveira in Portugal; Valdegoba in Spain; Payre, Rochers-de-Villeneuve, Rochelot, Les Pradelles, Grotte de la Tour and Grotte du Bison in France (op. cit. [116,117,49]); or Eel Point in the UK [118], among others, cf. [119]). The ubiquity and abundance of these species within assemblages yielding human remains does not preclude the role of other large carnivores as canids or large felids. It only reflects the fact that extinct hyenas were the only large carnivores in Africa, Europe and Asia that regularly accumulated dense concentration of bones, especially in cave sites, but does not imply that they were the greatest consumers of Plio-Pleistocene hominins [120].

### Hominins as prey or carrion?

Although we can conclude that the human femur was eaten by a hyena, we do not know how and by whom this bone was brought into the cave. Except in a few cases (see above in the introduction), the presence of carnivore tooth-marks on human remains is not sufficient to demonstrate predation, as they could have resulted from post-mortem consumption by scavengers.

At GH, other human remains are mainly cranial and vertebral elements devoid of any legible surface modifications. Nevertheless, the abundance of human fossils (NISP = 0.6%), at least as common as those of zebras, gelada baboons, warthogs, or some antelopes, and their scattering throughout the abundant carnivore refuse (see above Fig 2), may support the hypothesis of hominins as a possible common resource for carnivores. However we must be cautious, as many post-depositional processes occurred in the cave, affecting the spatial information and the understanding of bone accumulation processes.

Concerning the GH femur diaphysis, two scenarios are conceivable. If the carnivore that collected the bone also consumed it, then the hyena appears to be the most likely collector, as a hunter or as a scavenger. Alternatively, if the collector was not the consumer, then various accumulators may be involved. Although hominins belonged to a size class potentially threatened by hyena (*Crocuta* and *Hyaena*) or large-felid attacks (*Panthera* and potentially *Homotherium*)–baboons, bears or jackals being unlikely human hunters–[1,35,84], all these species and even other small collectors like porcupines could be potential scavengers, collecting the bone from a carcass and bringing it to the cave for storage and consumption.

## Conclusion

After the specimen from Aïn Maarouf, the ThI94-UA28-7 is the second human femur discovered in a Middle Pleistocene context in Morocco. Its characteristics match those of other early Middle Pleistocene *Homo*. It represents the first evidence of the consumption of human remains by carnivores at GH. In contrast, in the nearby, sub-contemporaneous site of GDR, the presence of a rich accumulation of animals hunted and consumed by humans [54] associated with an abundant Acheulean industry, points to hominins as effective predators, quite capable of evicting other carnivores from their habitats. Indeed, at GDR only one human individual was recorded (unpublished) despite a carnivore spectrum similar to that of GH. Similarly, other but more recent Middle Palaeolithic sites show occasional hunting and/or exploitation of large carnivores by hominins [121,122,36,38], highlighting human capacities to have successful confrontations with large carnivores.

During the Middle Pleistocene of North Africa, humans and carnivores competed for the same prey and natural shelters, resulting in a close proximity that could lead to many forms of interactions (see Introduction). Thomas-GH is a good example of one particular type of relationship: ‘Hominins as prey *(or carrion)* of carnivores’ [11]. Indeed, the contrasting evidence between the two Moroccan sites of GH and GDR shows that the status of hominins in the Ancient Palaeolithic food chain could alternate between carrion and/or prey, scavenger and/or predator, depending on the circumstances rather than on their abilities.
